# The location of the centre of the proximal quadriceps tendon in kinematically aligned total knee arthroplasty is not associated with poor outcome scores or symptomatic patellar instability

**DOI:** 10.1002/jeo2.70075

**Published:** 2024-11-13

**Authors:** Daniel Razick, Muzammil Akhtar, Stephen M. Howell, Alexander J. Nedopil, Maury L. Hull

**Affiliations:** ^1^ College of Medicine California Northstate University Elk Grove California USA; ^2^ Department of Biomedical Engineering University of California Davis California USA; ^3^ Orthopädische Klinik König‐Ludwig‐Haus Lehrstuhl für Orthopädie der Universität Würzburg Würzburg Germany; ^4^ Department of Orthopedic Surgery University of California Davis California USA; ^5^ Department of Mechanical Engineering University of California Davis California USA

**Keywords:** kinematics, outcomes, patellofemoral joint, quadriceps line of pull, tendon, trochlea

## Abstract

**Purpose:**

A previous study on osteoarthritic knees found that the average position of the centre of the proximal quadriceps tendon (PQT) was 9 mm lateral from the native trochlear groove. In patients with lateral patellar facet osteoarthritis, which indicates patellofemoral instability, the average location was 21 mm. The researchers suggested that a position more lateral than 20 mm might lead to poor outcomes after kinematically aligned total knee arthroplasty (KA TKA)—the current study aimed to test this hypothesis.

**Methods:**

The study involved all patients (*n* = 302) who underwent KA TKA (*n* = 313) in 2019, had a post‐operative long‐leg scanogram and knee computed tomography scan, and completed a 2‐year questionnaire. An evaluator measured the location of the PQT relative to the centre of the distal prosthetic trochlear groove. A Spearman's rank correlation coefficient analysis determined whether there was an association between the location of the PQT and the Forgotten Joint Score (FJS) and Oxford Knee Score (OKS) at 2 years.

**Results:**

The mean location of the PQT was 11 ± 8 mm (range, −2 medial to 36 mm lateral), with 16% (*N* = 46) of the KA TKAs having a more lateral location than 20 mm. The location of the PQT was not associated with the FJS (*r* = −0.0349, *p* = 0.7281) and OKS (*r* = −0.0641, *p* = 0.9009)—no patient response indicated symptoms or operative treatment for patellofemoral instability.

**Conclusion:**

Even though 16% of patients with a KA TKA had a more lateral location than 20 mm, there is no reason to measure the centre of the PQT relative to the distal prosthetic groove. This is because the location did not show any association with the 2‐year FJS and OKS nor had any patient experienced patellofemoral instability.

**Level of Evidence:**

IV.

**Abbreviations**
aHKAA: arithmetic hip–knee–ankle angle
FJS: Forgotten Joint Score
ICC: intra‐class correlation coefficient
JLO: joint line obliquity
KA: kinematic alignment
KA TKA: kinematically aligned total knee arthroplasty
LDFA: lateral distal femoral angle
MA: mechanically aligned
MPR: Multiplane Reconstruction Tool
MPTA: medial proximal tibial angle
OKS: Oxford Knee Score
PQT: proximal quadriceps tendon
QT: quadriceps tendon
SD: standard deviation
TKA: total knee arthroplasty

## INTRODUCTION

Complications in the patellofemoral joint can occur after total knee arthroplasty (TKA), including patellar instability, anterior knee pain, patellar crepitus, patellar component loosening, wear, and soft‐tissue impingement [2, 4, 7, 18]. Identifying the anatomical features contributing to extensor mechanism malalignment might reduce the risk of these complications, as they often require revision surgery.

A previous computed tomography (CT) scan study of osteoarthritic knees reported that the mean centre of the proximal quadriceps tendon (PQT) was 9 mm (standard deviation [SD] 7.7 mm) lateral to the native trochlear groove. In those with lateral patellar facet osteoarthritis, the mean location was 21 mm lateral, suggesting lateral patella maltracking. The authors hypothesized that a location more lateral than 20 mm after kinematically aligned (KA) TKA might lead to poor clinical outcomes and patellofemoral instability since the KA technique, which resurfaces the pre‐arthritic knee, does not change the QT location and Q‐angle [10, 11].

This hypothesis might not be valid as the prior study used a mechanical axis reference frame to locate the centre of the PQT [24]. Because mechanical alignment changes the knee's pre‐arthritic Q‐angle and joint line orientation, the kinematic axis reference plane should be used to measure the location of the PQT in KA TKA [8, 10, 16, 22].

No research studies have been conducted to determine the location of the centre of the PQT relative to the distal prosthetic trochlear groove in patients treated with KA TKA. Hence, our study aimed to address two main questions: (1) What is the mean location of the PQT after KA TKA, and what proportion is the location more than 20 mm? (2) Is there an association between the location and the 2‐year Forgotten Joint Score (FJS) and Oxford Knee Score (OKS), and symptomatic patellofemoral instability?

## MATERIALS AND METHODS

An Institutional Review Board approved a retrospective analysis of de‐identified data (Pro00075103) obtained from a database of prospectively archived patient records and radiographs. In 2019, the senior author performed 415 KA TKAs. Each patient fulfilled the Centres for Medicare & Medicaid Services guidelines for medical necessity for TKA treatment and had (1) Kellgren–Lawrence Grade III to IV osteoarthritis; (2) any severity of varus or valgus deformity; and (3) any severity of flexion contracture. Excluded were patients who had prior fractures of the knee treated with open‐reduction internal fixation, inflammatory or septic arthritis, and lower extremity neurologic disorders.

The surgeon performed a cemented calliper‐verified KA TKA using a femoral component designed with a 6° prosthetic trochlear groove angle, an insert with intermediate medial conformity, and a flat lateral articular surface enabling posterior cruciate ligament retention, and patella resurfacing (GMK Sphere, Medacta International). The technique relied on manual instruments and calliper verification of the thickness of the bone resections, which has a negligible learning curve [19]. It aligns the femoral component within 0 ± 0.4 mm of the pre‐arthritic articular surface, which is more accurate than robotics [9].

On the day of discharge, each patient consented to an anterior–posterior, non‐weight‐bearing, long‐leg scanogram and a CT scan confined to the knee. The average radiation dose of 0.1–0.2 mSv is less than the 0.7 mSv reported for the conventional long‐leg radiograph [17]. For the scanogram, the technician standardized leg rotation to minimize projection and angular measurement errors by setting the internal–external rotation of the knee until each posterior condyle of the femoral component was partially visible on either side of the anterior flange of the femoral component [20].

Two years from the date of surgery, digital data collection software (Caresense) sent each patient a questionnaire via text and email asking them to fill out the FJS (100 best, 0 worst) and OKS (48 best, and 0 worst) and provide details on any concerns about knee function and list any operations on the knee after the index surgery. One investigator accessed five ‘people search’ websites to determine the whereabouts of non‐responding patients, who were contacted by phone when located.

### Radiologic method for measuring the location of the centre of the PQT

**Figure 1:**
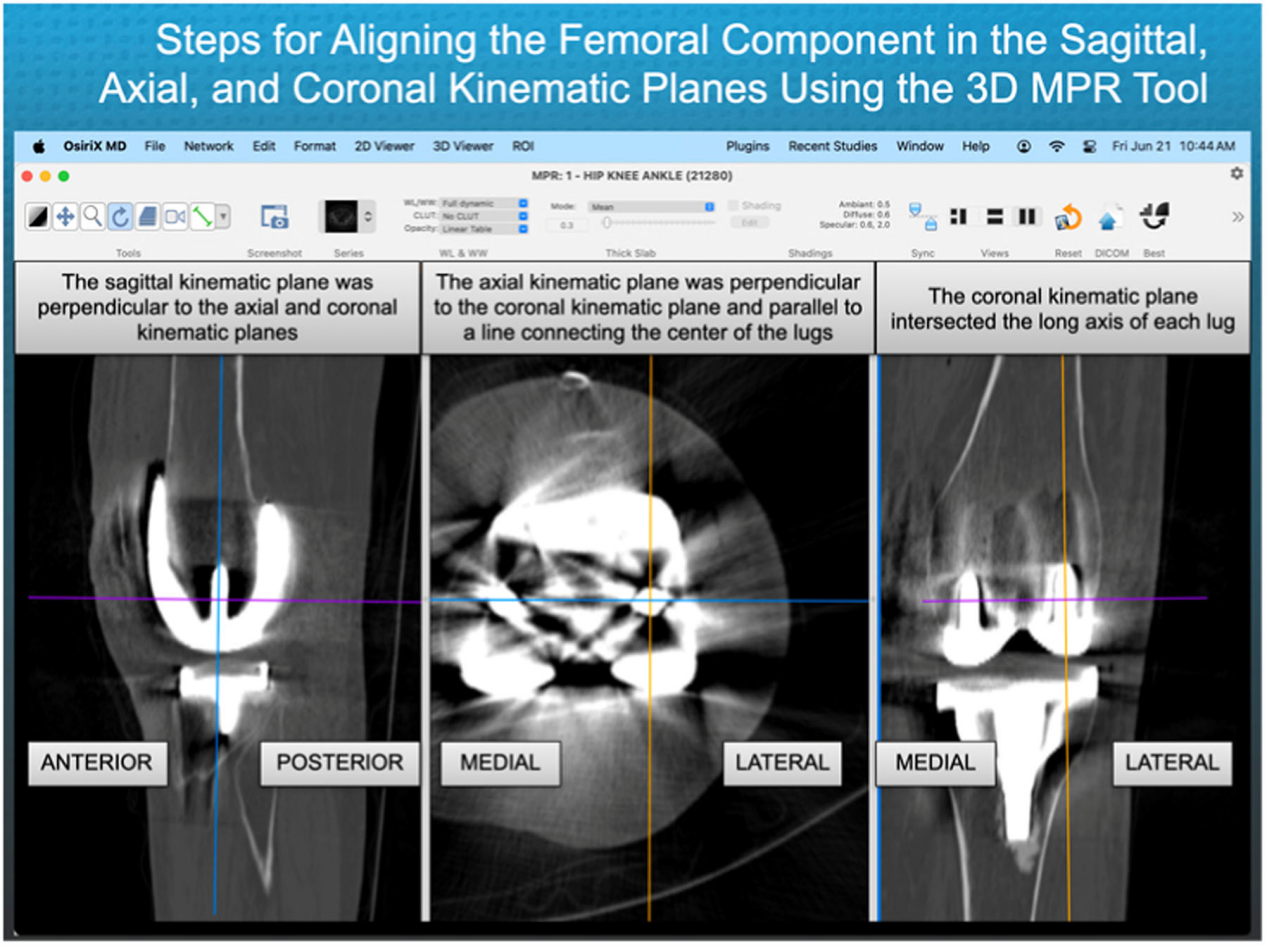
The image displays the menu bar containing the software settings for viewing the CT scan of the KA TKA. It demonstrates the steps for using the lugs of the femoral component to orient the knee in the sagittal, axial and coronal kinematic axis reference planes using the 3D multiplane (MPR) viewer. CT, computed tomography; KA TKA, kinematically aligned total knee arthroplasty; MPR, Multiplane Reconstruction Tool.

Two evaluators (DR and MA) used three‐dimensional image‐analysis software to measure the location of the centre of the PQT. They viewed the knee using the 3D Multiplane Reconstruction Tool (MPR) (OSIRIX MD, v.14.0.1, Pixmeo). The KA TKA was oriented in the kinematic reference frame by setting the coronal kinematic plane parallel to the long axis of both lugs, the axial kinematic plane parallel to a line connecting both lugs and perpendicular to the coronal kinematic plane, and the sagittal kinematic plane perpendicular to the coronal and axial planes (Figure 1).

**Figure 2:**
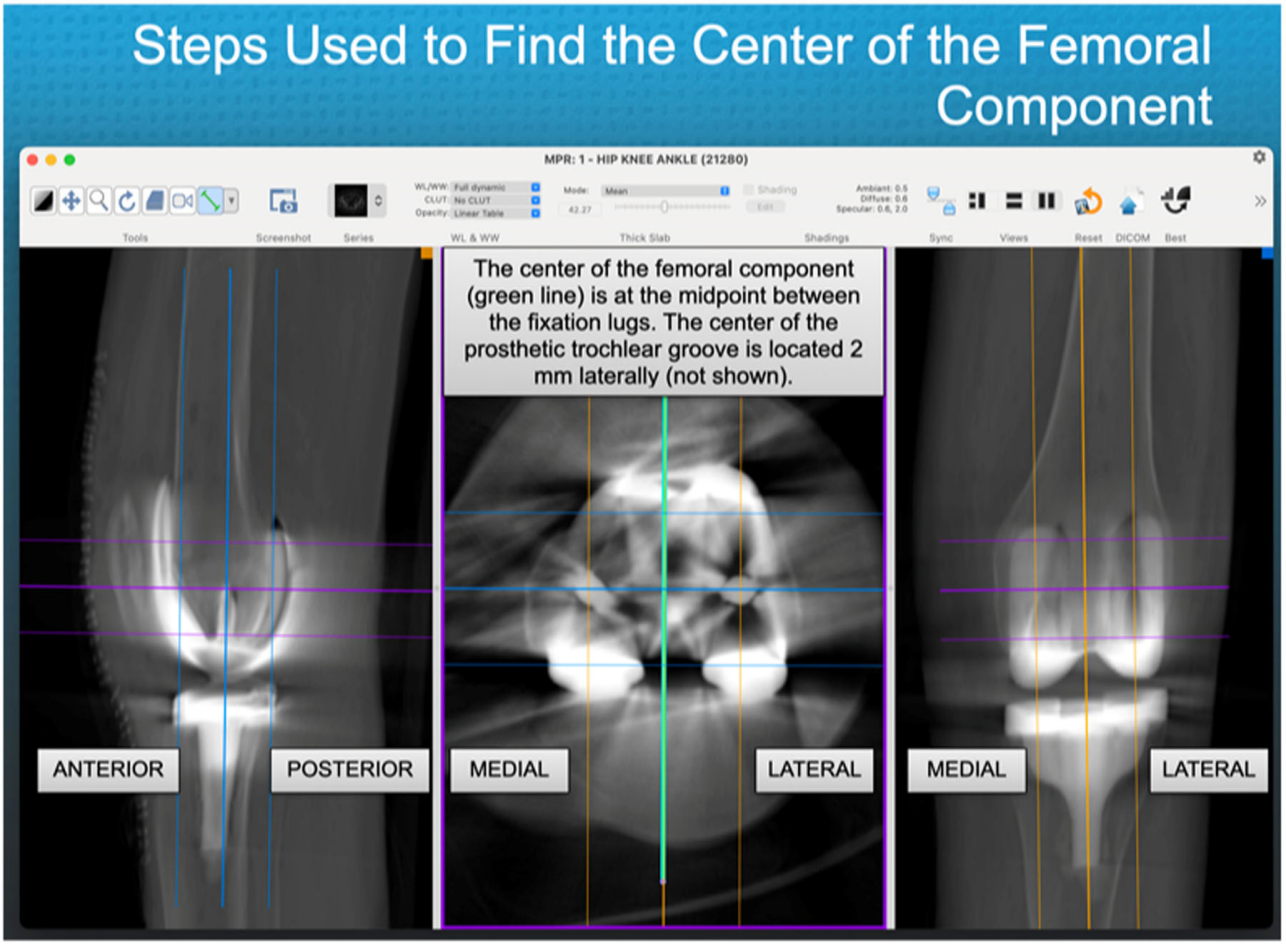
The image displays the menu bar containing the software settings for viewing the CT scan of the KA TKA. It also shows the process of drawing a vertical line (in green) on the axial image to indicate the centre of the femoral component. CT, computed tomography; KA TKA, kinematically aligned total knee arthroplasty.

**Figure 3:**
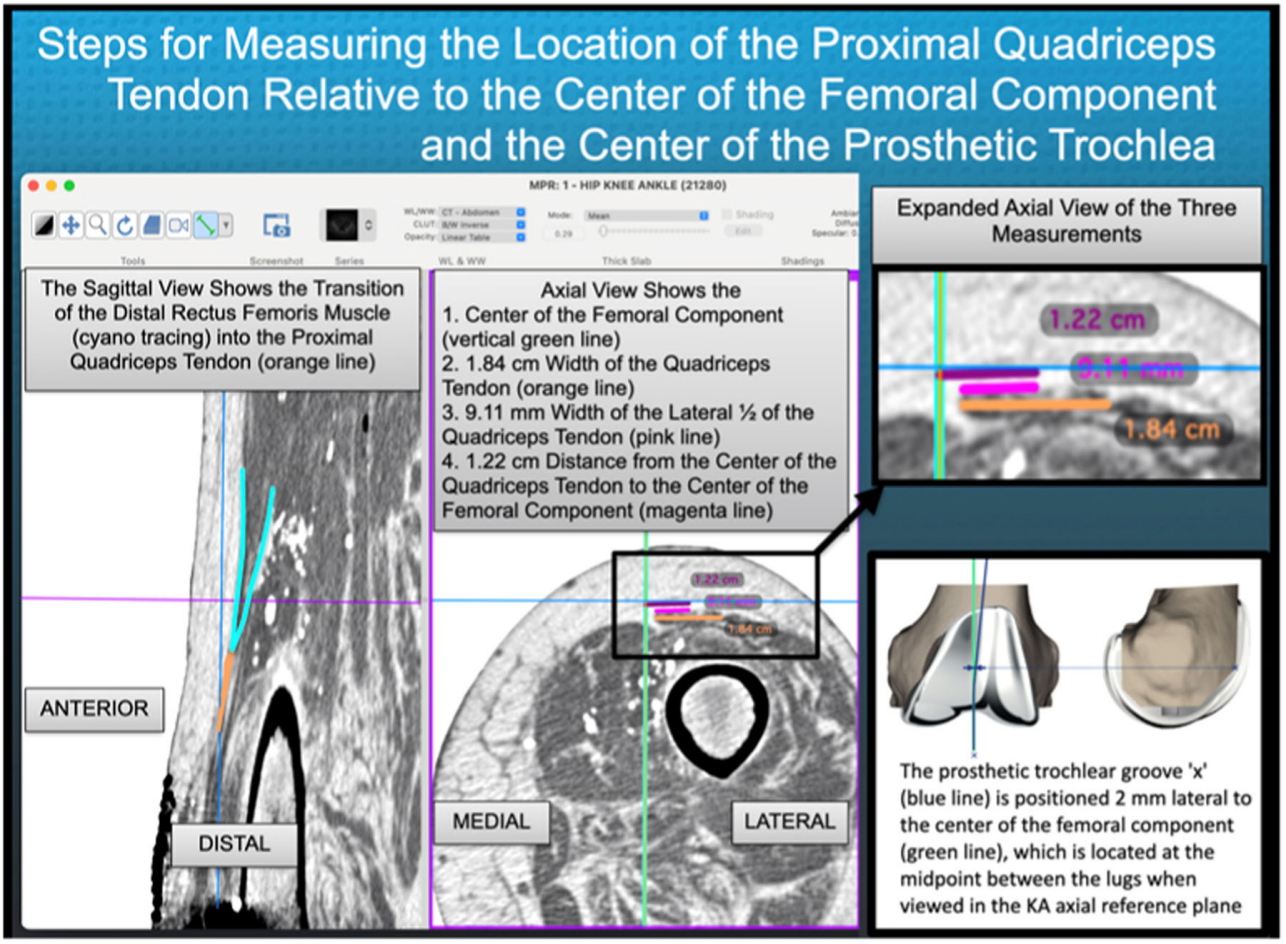
The image shows the menu bar with the software settings for viewing the knee in the sagittal and axial kinematic axis reference planes. It is used to measure the location of the proximal quadriceps tendon (PQT) relative to the centre of the femoral component and compute the location relative to the prosthetic trochlear groove. The image demonstrates the steps for identifying the transition from the distal rectus femoris muscle to the PQT in the sagittal plane (left panel) and for identifying the transverse width (orange line) of the most proximal portion of the PQT in the axial plane (centre panel).

The evaluator took the following steps to measure the location of the centre of the PQT relative to the distal prosthetic trochlear groove. In the axial view, a green vertical line was drawn at the centre of the femoral component, midway between the femoral fixation lugs and then copied (Figure 2). When viewing the axial plane, the evaluator scrolled down from the rectus femoris muscle until the most PQT cross‐section was visible. A region of interest tool drew a line along the transverse width of the PQT; the width was measured, and the midpoint of the width was identified, which defined the centre of the QT. The line marking the centre of the femoral component was pasted. The horizontal distance between the centre of the QT and the centre of the femoral component was measured. Based on the manufacturer's analysis of all the femoral component sizes, which showed that the prosthetic trochlear groove was 2 mm lateral to the centre of the femoral component, 2 mm was subtracted from this value to determine the location of the PQT relative to the distal prosthetic trochlear groove (Figure 3). The location of the PQT relative to the distal prosthetic trochlear groove was normalized by multiplying it by the transverse width of the patient's femoral component and dividing it by 68 mm, which is the width of a midsize (i.e., size 4) femoral component.

### Statistical analysis

The authors used statistical software to analyse the data (JMP Pro, 17.2.0, JMP Statistical Discovery LLC), with *p* < 0.05 indicating a significant difference. The mean ± SD and median and interquartile range described normal and non‐normal random variables, respectively. A Spearman's rank correlation coefficient analysis determined whether there was an association between the location of the centre of the PQT and FJS and OKS at 2 years.

Two statistical analyses determined the consistency of measuring the medial–lateral location of the PQT relative to the distal prosthetic groove of the femoral component. The first analysis computed the intra‐observer and the inter‐observer variability intra‐class correlation coefficient (ICC) using measurements made on 12 randomly selected TKAs by three observers [1]. The second analysis quantified the precision of the measurement (i.e. repeatability) by computing the square root of the pooled variance from three measurements of the location of the QT made on alternating days on 12 randomly selected TKAs by three observers. The intra‐ and inter‐observer ICCs and precision were 0.95, 0.94 and 1.5 mm, respectively.

## RESULTS

Out of an initial group of 415 KA TKAs (403 patients) considered eligible for the study, 35 were excluded because they did not have a post‐operative CT scan, and 67 were excluded because the patient did not fill out the post‐operative questionnaire. This left 313 KA TKAs (302 patients) for the present study who filled out the questionnaire with an average follow‐up period of 30 ± 5 months. Table 1 compares patient demographics and preoperative characteristics (sex, body mass index, maximum knee flexion and clinical scores) for the included and excluded patients.

**Table 1:** The table lists the preoperative characteristics and function scores for the included and excluded KA TKAs in the present study and the results of the statistical analysis of the differences between groups.

Preoperative characteristics	Included subjects with CT scan and questionnaire	Excluded subjects because of no CT scan	Excluded subjects because of unfilled questionnaire	*p*
Number of KA TKAs	313	35	67	
Age	69 ± 8 years	69 ± 8 years	72 ± 9 years	**0.039**
Sex	171 females, 131 males	19 females, 15 males	42 females, 25 males	0.767
Body mass index	30 ± 5 kg/m^2^	31 ± 7 kg/m^2^	30 ± 5 kg/m^2^	0.510
Knee range of motion	105 ± 10°	102 ± 12°	101 ± 13°	**0.022**
Alignment (−varus/−valgus)	−4 ± 11°	1 ± 13°	0 ± 13°	**0.006**
Oxford Knee Score (48 is best, 0 is worst)	23 ± 8 points	23 ± 9 points	20 ± 7 points	0.109
Knee Society Score Knee (100 is best, 0 is worst)	37 ± 13 points	39 ± 16 points	38 ± 12 points	0.627
Knee Society Score Functional (100 is best, 0 is worst)	54 ± 20 points	53 ± 17 points	47 ± 16 points	**0.017**

*Note*: Values reported as mean ± standard deviation. The bold values are less than 0.05 and hence they indicate the differences were significantly different.

Abbreviations: CT, computed tomography; KA TKA, kinematically aligned total knee arthroplasty.

**Figure 4:**
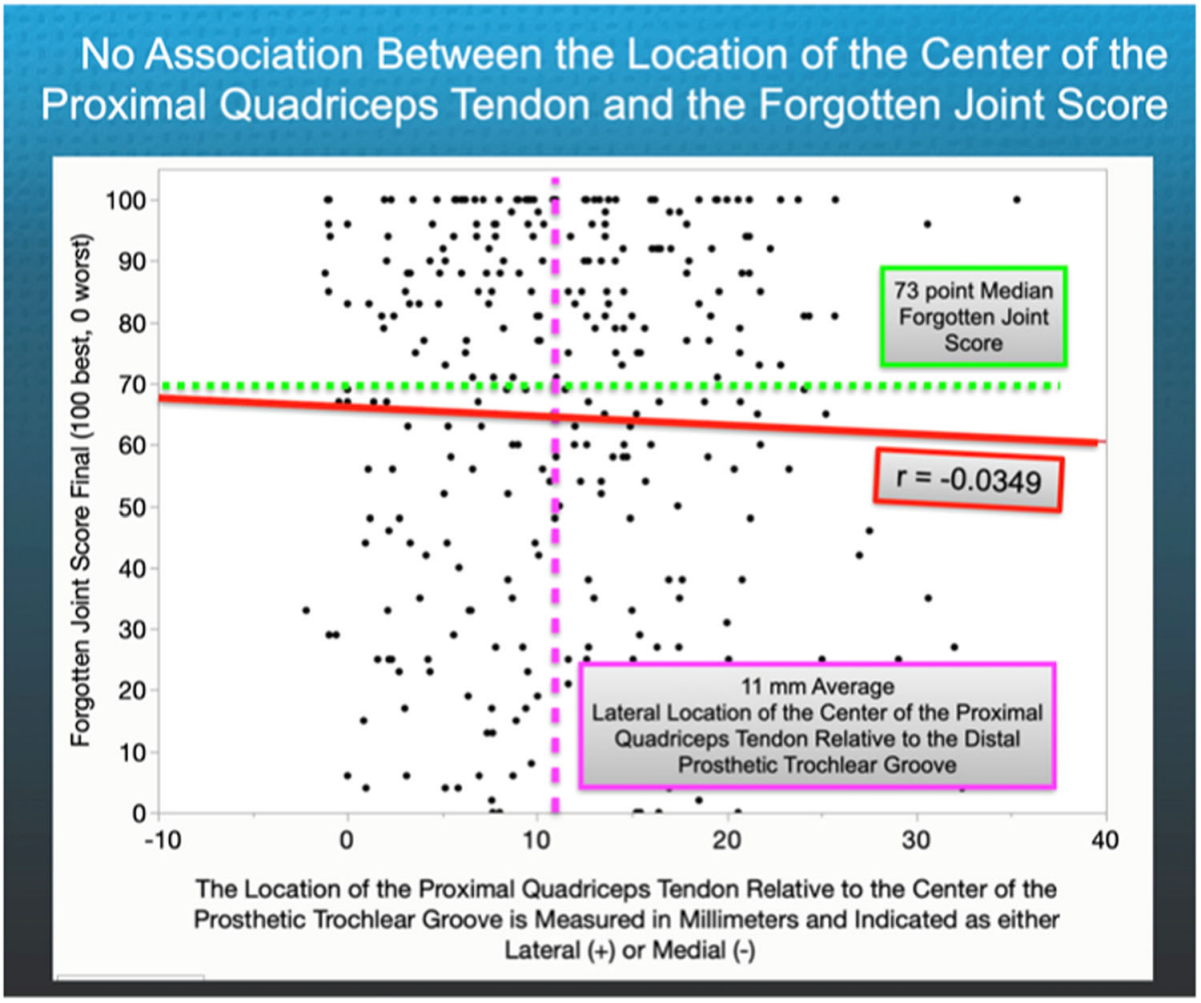
Based on the scatterplot analysis, there is no clinically meaningful correlation between the medial–lateral distance of the centre of the proximal quadriceps tendon relative to the distal prosthetic trochlear groove and the Forgotten Joint Score (where 100 is the best score and 0 is the worst score) (*r* = −0.0349, *p* = 0.7281).

**Figure 5:**
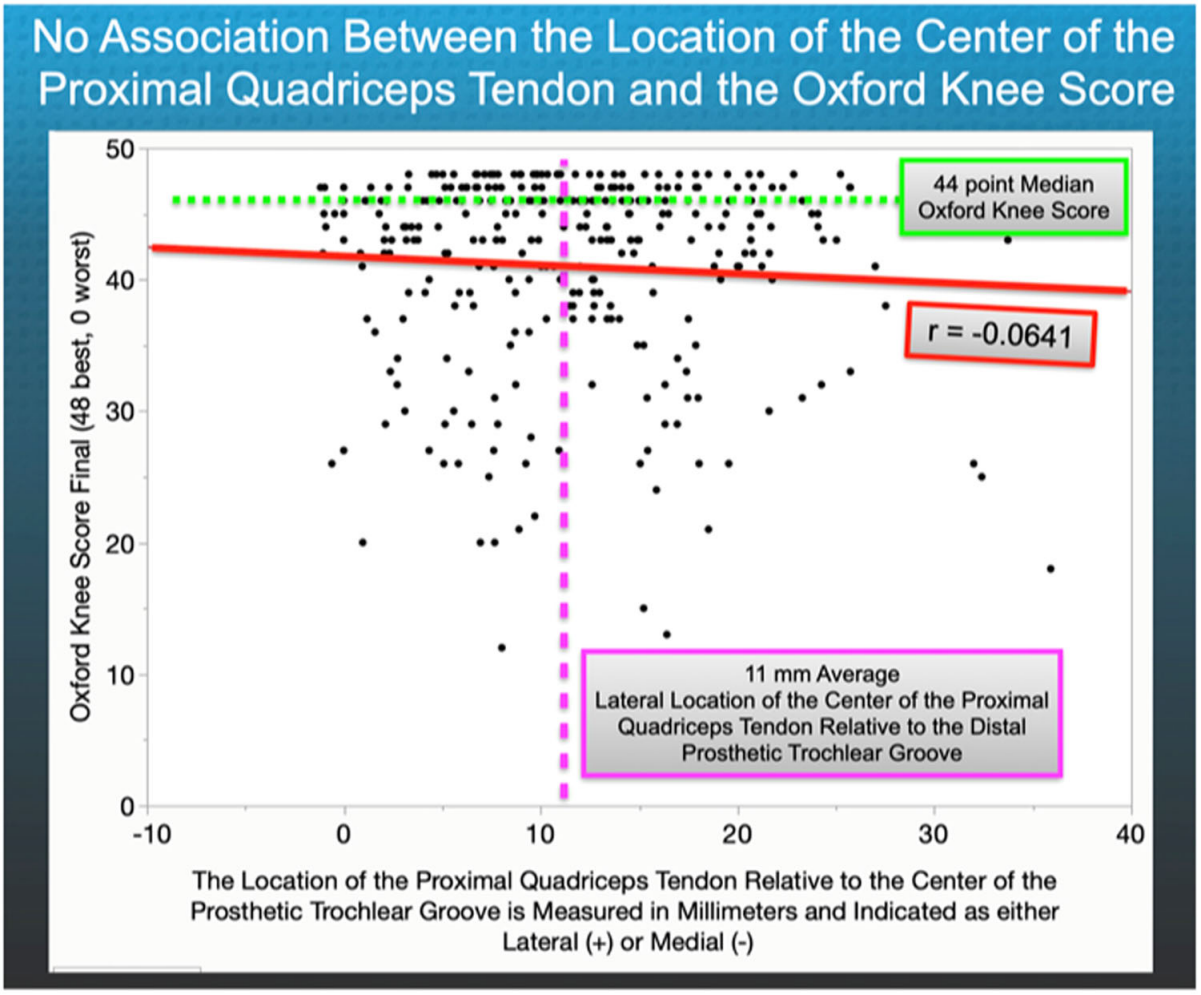
Based on the scatterplot analysis, there is no clinically meaningful correlation between the medial–lateral distance of the centre of the proximal quadriceps tendon relative to the prosthetic trochlear groove and the Oxford Knee Score (where is the best score, 0 is the worst score) (*y*‐axis) (*r* = −0.0641, *p* = 0.9009).

The mean location of the PQT was 11 ± 8 mm (range, −2 medial to 36 mm lateral). Sixteen per cent (*N* = 46) of the KA TKAs had a more lateral location than 20 mm. The location of the PQT did not show any correlation with the FJS (*r* = −0.0349, *p* = 0.7281) and OKS (*r* = 0.0641, *p* = 0.9009) (Figures 4 and 5). None of the responses on the questionnaire indicated that any patient had experienced symptoms or received operative treatment for patellofemoral instability. Table 2 lists the post‐operative radiologic analysis of the limb and component alignment and 2‐year function scores.

**Table 2:** The table lists the post‐operative radiologic analysis of the limb and component alignment and 2‐year function scores.

	313 KA TKAs
**Post‐operative radiographic analysis**	Angle^a^
Hip knee ankle angle (<180° is varus)	179 ± 3° (170−186°)
Distal lateral femoral angle (<90° is valgus)	87 ± 2° (79−93°)
Proximal medial tibial angle (<90° is varus)	86 ± 2° (79−93°)
**Post‐operative patient‐reported outcome measures**	Points^b^
Forgotten Joint Score (100 is best, 0 is worst)	73 [38–92]
Oxford Knee Score (48 is best, 0 is worst)	44 [38–47]

Abbreviation: KA TKA, kinematically aligned total knee arthroplasty.

^a^ Values reported as mean ± standard deviation (range).

^b^ Values reported as median [interquartile range].

## DISCUSSION

The key finding from this study on KA TKA is that, on average, the centre of the PQT is located 11 ± 8 mm laterally (range, −2 mm medial to 36 mm lateral) relative to the distal prosthetic groove. The study also found that even though 16% of the KA TKAs had a more lateral location than 20 mm, measuring the location of the PQT is unnecessary as it did not show any correlation with the 2‐year FJS, OKS and no patients reported symptoms or reoperations for patellofemoral instability.

There are at least two reasons why the position of the PQT relative to the distal prosthetic trochlear groove, when measured in a kinematic reference frame, is not a reliable indicator of the FJS, OKS and patellofemoral instability following KA TKA. One is that patellar tracking is influenced by factors other than the location of the PQT, although there is no difference in trochlear morphology between the sexes [15]. First, there are notable differences in the morphology and angle primarily of the anterior portion of the prosthetic trochlea groove as compared to the native trochlea, the morphology and thickness of the resurfaced patella versus the native patella, and the tension in the medial retinacular ligament after arthrotomy repair versus the intact knee [10, 16, 22, 23]. Second, the native groove orientation is significantly altered after TKA, regardless of the alignment strategy used. KA produces the lowest number of outliers compared to functional alignment, mechanical alignment and adjusted mechanical alignment, but a substantial number remains [13].

There are several reasons for the low risk of patellar instability in 313 KA TKAs performed with manual instruments, regardless of the severity of the preoperative varus‐valgus deformity and flexion contracture and following the principle of complete post‐operative correction to the pre‐arthritic alignment. For instance, a 13‐year follow‐up of a randomized control trial reported that KA TKA had similar reoperations and complications to mechanically aligned (MA) TKA [5]. A 7‐year follow‐up by the Australian and New Zealand combined arthroplasty registry revealed that the most common reasons for revision in KA TKA with patient‐specific guides were patellar erosion of an unresurfaced patella and patellar maltracking, which has been associated with excessive flexion of the femoral component due to a poor fit of the femoral guide [14, 18]. Excessive flexion of the femoral component is minimized by using manual instruments, which results in an average of 5° less flexion compared to those femoral components placed with patient‐specific instrumentation [6]. In addition, a systematic review and clinical and cadaveric studies demonstrated that KA TKAs showed better restoration of patellar tracking than the MA TKA technique [3, 11, 12, 21]. The better patellofemoral kinematics is explained by KA restoring the pre‐arthritic Q‐angle, while MA increases the Q‐angle in 70% of pre‐arthritic knees with constitutional varus alignment [16].

## CONCLUSION

Even though 16% of patients with a KA TKA had a more lateral location than 20 mm, there is no reason to measure the centre of the PQT relative to the distal prosthetic groove. This is because the location did not show any association with the 2‐year FJS and OKS nor had any patients reported symptoms or reoperations for patellofemoral instability.

## AUTHOR CONTRIBUTIONS

All authors contributed to the study conception, design, material preparation, data collection, analysis and writing. All authors read and approved the final manuscript.

## FUNDING INFORMATION

This research received no external funding.

## CONFLICT OF INTEREST STATEMENT

Stephen M. Howell and Alexander J. Nedopil receive consulting fees and royalties from Medacta International (Castel San Pietro, Switzerland, www.medacta.com). Maury L. Hull receives research support from Medacta International (Castel San Pietro, Switzerland, www.medacta.com). The other authors declare no conflict of interest.

## ETHICS STATEMENT

This study was approved by the Institutional Review Board. The board approved a retrospective analysis of de‐identified data (Pro00075103) obtained from a database consisting of prospectively archived patient records and radiographs.

## Data Availability

The data sets used and/or analysed during the current study are available from the corresponding author on reasonable request.
